# Effects of stent shape on focal hemodynamics in intracranial atherosclerotic stenosis: A simulation study with computational fluid dynamics modeling

**DOI:** 10.3389/fneur.2022.1067566

**Published:** 2022-12-13

**Authors:** Haipeng Liu, Yu Liu, Bonaventure Y. M. Ip, Sze Ho Ma, Jill Abrigo, Yannie O. Y. Soo, Thomas W. Leung, Xinyi Leng

**Affiliations:** ^1^Department of Medicine and Therapeutics, The Chinese University of Hong Kong, Hong Kong, Hong Kong SAR, China; ^2^Department of Imaging and Interventional Radiology, The Chinese University of Hong Kong, Hong Kong, Hong Kong SAR, China; ^3^Research Centre for Intelligent Healthcare, Coventry University, Coventry, United Kingdom

**Keywords:** intracranial atherosclerotic stenosis (ICAS), stent geometry, in-stent restenosis (ISR), hemodynamics, computational fluid dynamics (CFD), wall shear stress (WSS), low-density lipoprotein (LDL)

## Abstract

**Background and aims:**

The shape of a stent could influence focal hemodynamics and subsequently plaque growth or in-stent restenosis in intracranial atherosclerotic stenosis (ICAS). In this preliminary study, we aim to investigate the associations between stent shapes and focal hemodynamics in ICAS, using computational fluid dynamics (CFD) simulations with manually manipulated stents of different shapes.

**Methods:**

We built an idealized artery model, and reconstructed four patient-specific models of ICAS. In each model, three variations of stent geometry (i.e., enlarged, inner-narrowed, and outer-narrowed) were developed. We performed static CFD simulation on the idealized model and three patient-specific models, and transient CFD simulation of three cardiac cycles on one patient-specific model. Pressure, wall shear stress (WSS), and low-density lipoprotein (LDL) filtration rate were quantified in the CFD models, and compared between models with an inner- or outer-narrowed stent vs. an enlarged stent. The absolute difference in each hemodynamic parameter was obtained by subtracting values from two models; a normalized difference (ND) was calculated as the ratio of the absolute difference and the value in the enlarged stent model, both area-averaged throughout the arterial wall.

**Results:**

The differences in focal pressure in models with different stent geometry were negligible (ND<1% for all cases). However, there were significant differences in the WSS and LDL filtration rate with different stent geometry, with ND >20% in a static model. Observable differences in WSS and LDL filtration rate mainly appeared in area adjacent to and immediately distal to the stent. In the transient simulation, the LDL filtration rate had milder temporal fluctuations than WSS.

**Conclusions:**

The stent geometry might influence the focal WSS and LDL filtration rate in ICAS, with negligible effect on pressure. Future studies are warranted to verify the relevance of the changes in these hemodynamic parameters in governing plaque growth and possibly in-stent restenosis in ICAS.

## Introduction

Intracranial atherosclerotic stenosis (ICAS) is an important cause of ischemic stroke and transient ischemic attack (TIA) (1). Stenting is an optional treatment for patients with symptomatic ICAS who failed medical treatment, i.e., with recurrent stroke despite medical treatment (2). However, there is a considerable incidence of in-stent restenosis (ISR) after stenting. The average rate of ISR reaches 15% with a mean follow-up of 18 months, in a recent systematic review of 52 studies (3).

Previous studies have indicated that both biochemical (e.g., dyslipidemia) and geometric (e.g., arterial tortuosity, stenosis, size of stent) features can be risk factors of further development of the atherosclerotic lesion and subsequent ISR, where focal hemodynamics may play an important role in linking them to ISR. Relevant studies in coronary and carotid arteries have revealed that geometric features of the stented artery, such as the arterial diameter (4), vessel curvature (4–6), adjacent bifurcation angle (7) and the lesion length (8), as well as the size and shape of the stent (9, 10), can significantly influence focal hemodynamics. On the other hand, the hemodynamic features, such as high pressure and low wall shear stress (WSS) can increase the filtration rate of low-density lipoprotein (LDL) in vascular endothelium, which can accelerate the growth of atherosclerotic plaques and increase the risk of ISR (11).

Computational fluid dynamics (CFD) simulation provides a non-invasive method to estimate focal hemodynamic parameters in vascular diseases, for instance, the quantification of pressure and WSS (12). It can also provide insights into the behavior of LDL in different arterial or flow conditions, e.g., the LDL concentration adjacent to the inner arterial wall surface, when lower WSS and increased LDL concentration were observed with larger bending angles in a coronary artery model (6). Further, the filtration rate of LDL on the arterial wall could also be estimated based on the focal hemodynamic parameters (e.g., pressure, WSS) obtained in the CFD models, using a 3-pore filtration model (11, 13). In studies of coronary and aortic arteries, risky areas of atherosclerotic plaque growth predicted by this method were consistent with that observed in clinical and imaging studies (11, 14). However, intracranial arteries are different from extracranial arteries in histology and geometry, with the former having thinner artery wall, more curvatures and higher tortuosity in the arterial tree (12). Therefore, findings from previous CFD studies on extracranial arteries may not be directly applicable to intracranial arteries.

By far, relevant data are scarce to estimate the effects of stent geometry on focal hemodynamic parameters in ICAS, which could help understand and predict the development of atherosclerotic lesion after stenting and subsequent ISR. In this pilot study, we aimed to investigate the associations between stent shapes and focal hemodynamics in ICAS using CFD simulations in an idealized intracranial arterial model and patient-specific ICAS models, with manually manipulated stents of different shapes.

## Methods

We simulated different stent shapes and conducted static CFD analyses on an idealized intracranial arterial model and three patient-specific ICAS models. We also simulated different stent shapes and performed transient CFD analyses in a patient-specific ICAS model. The patient-specific ICAS models were obtained from the SOpHIA study (Stroke Risk and Hemodynamics in Intracranial Atherosclerotic Disease), a cohort study to investigate cerebral hemodynamics in patients with symptomatic ICAS, using computed tomography angiography (CTA)-based CFD models (15). The local institutional review board approved the study and all patients provided informed consent.

In each of the idealized and patient-specific models, we virtually placed stents with three manually manipulated shapes across the stenotic lesion, namely the “enlarged,” “inner-narrowed” and “outer-narrowed” shapes, and conducted CFD modeling in each scenario. We quantified focal pressure, WSS and the filtration rate of LDL on arterial wall in the CFD models. In each case, we compared these parameters obtained from the stents of three geometric variations. More detailed methodology is described below and in our previous works (11, 15–18).

### Idealized model and patient-specific models for static simulation

#### Arterial models and manually manipulated stent shapes

**Figure 1:**
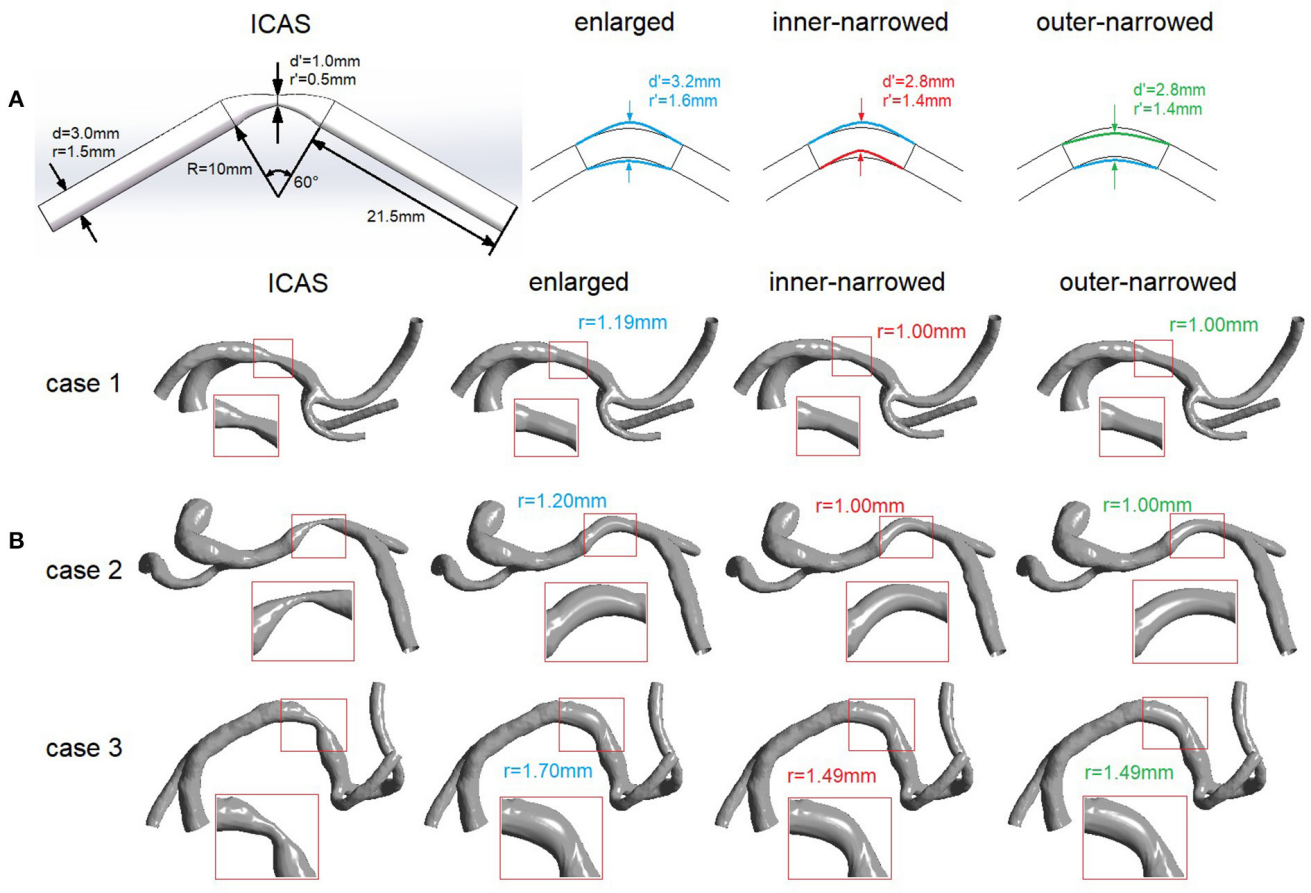
Idealized **(A)** and patient-specific **(B)** ICAS and stenting models. d, diameter; r, radius; R, radius of curvature. **(A)** Left: Idealized geometry of an intracranial artery with ICAS. Right: simulated variations of the vessel geometry after stenting, with 3 different stent shapes. **(B)** Geometric models of ICAS lesions in M1 segment of MCA in 3 patients, and the simulated variations of the vessel geometry after stenting, with 3 different stent shapes. The arterial segment adjacent to the ICAS lesion that is virtually stented is magnified with red rectangle in each scenario.

As shown in Figure 1A, we built an idealized tube model of a stenosed artery with the radius of 1.5 mm, similar as that of middle cerebral artery (MCA), and 60°curved at the middle section with a 10 mm radius of curvature to simulate the curvature of intracranial arteries. The radius was 0.5 mm at stenotic throat (i.e., the narrowest location), with a 67% luminal stenosis according to the Warfarin-Aspirin Symptomatic Intracranial Disease (WASID) method (19).

Three variations of stent shapes were developed (Figure 1A). First, the radius of the middle cross-section (i.e., the cross-section at stenotic throat, marked with two blue arrows) was enlarged to 1.6 mm and lofted to the ends of curved segment while adjacent segments beyond the both ends were unaffected, forming the “enlarged” variation. Similarly, we changed the radius of the middle cross-section (marked with two red or green arrows) to 1.4 mm, then moved its center upward or downward where the cross-sectional circle was tangent to the enlarged arterial wall, forming two variations named “inner-narrowed” and “outer-narrowed” to indicate the narrowed side.

The patient-specific models of internal carotid artery (ICA)-MCA-anterior cerebral artery (ACA) branches were reconstructed from the CTA images using the software MIMICS 14.0 (Materialize Europe, Belgium), in 3 patients with a symptomatic ICAS lesion respectively located at proximal, middle and distal 1/3 of M1 segment of MCA (rectangles in Figure 1B). The degree of luminal stenosis was respectively 80, 63, and 67% in these cases (cases 1–3 in Figure 1B), measured by the WASID method (19). Stents with the three different shapes were virtually placed across the ICAS lesion, similarly as in the idealized model. In the “enlarged” variation, the area of the cross-section at the stenotic throat after stenting was larger than the cross-sections at both ends. In the “inner-narrowed” and “outer-narrowed” variations, the radius of the cross-section was decreased by approximately 0.2 mm and moved tangent to the outer and inner side of the curvature of the enlarged focal artery segment (Figure 1B). Compared with the enlarged stent model, the inner-narrowed and outer-narrowed models were shrunk at the inner and outer side of the curvature, respectively. At the stenotic throat, the inner-narrowed and outer-narrowed models had an identical cross-sectional area after stenting.

#### Meshing and CFD simulation

In each case, CFD modeling was separately conducted in the scenarios with “enlarged”, “inner-narrowed” and “outer-narrowed” stent shapes. The geometric models were meshed and simulated in Ansys ICEM-CFX 15.0 (ANSYS Inc., Canonsburg, PA), with maximum element size of 0.25 mm globally and 0.1 mm at inlet and outlets as validated in the SOpHIA study (15, 16, 18).

For the idealized model, the boundary conditions were 110 mmHg at the inlet and area-averaged velocity of 35 cm/s at the outlet. In patient-specific static models, 110 mmHg, 35 cm/s and 31 cm/s were applied at the ICA inlet, MCA outlet, and ACA outlet, as in our previous studies (16, 17). In all the simulations, we adopted the non-slip wall assumption and the convergence criterion of 10^−4^. Blood is a non-Newtonian fluid with shear-thinning properties. To avoid possible inaccuracy of Newtonian fluid model in WSS estimation (20), we adopted the Carreau-Yasuda non-Newtonian rheological model (11, 16).

#### Quantification of focal hemodynamic parameters

In each case, firstly we calculated the absolute differences in the hemodynamic parameters between the inner- or outer-narrowed stent models vs. the enlarged model. The absolute differences in the parameters were mapped on the inner- or outer-narrowed stent models.

Next, we obtained the mean value of each hemodynamic parameter throughout the arterial wall area in the entire model in each case, i.e., area-averaged value, separately in the CFD models built with the three stent shapes. To estimate the effects of stent shapes on focal hemodynamic parameters in the intracranial arteries, we calculated the normalized difference (ND) of each hemodynamic parameter between the inner- or outer-narrowed model and the enlarged model, as the absolute difference divided by the value measured on the enlarged model, both area-averaged throughout the whole artery wall, as shown in Equation 1. ND and V denote normalized difference and hemodynamic parameter value; subscript i denotes the inner- or outer-narrowed models, and R denotes the enlarged model (as a reference).


(1) $$\begin{array}{l} \begin{array}{c} {\text{N}\text{D}}_{\text{i}}=\;\frac{|{\text{V}}_{\text{i}}-{\text{V}}_{\text{R}}{|}_{\text{area}-\text{averaged}}}{{{\text{V}}_{\text{R}}}_{\text{area}-\text{averaged}}} \end{array} \end{array}$$

We also assessed the effects of stent shapes on the distribution of WSS in the outer and inner sides of the stented arterial segment. In each model, the surface of the stented segment (magnified in Figure 1) was approximately halved into the inner and outer sides of the curvature. The area-averaged WSS was calculated on the inner and outer sides of the stented segment, respectively. We then calculated the ratio of area-averaged WSS values obtained at the outer and inner sides of the stented segment in each model.

#### Calculation of LDL filtration rate

There are three pathways for the transport of LDL molecules across the endothelium into the arterial wall: transcytosis in vesicles, paracellular transport through the breaks in the tight junction strand, and leaky junctions associated with cell turnover or apoptosis, in which leaky junctions accounts for around 90% of LDL transport under convective conditions (21). Therefore, only the LDL filtration *via* leaky junctions was calculated in this pilot study using a simplified 3-pore model, which was the LDL filtration rate per one leaky junction multiplied by the local density of leaky junctions (11). The density of leaky junctions, i.e., the number of leaky junctions per area, was calculated from the WSS value, with more leaky junctions in low-WSS areas (22). The LDL filtration rate per one leaky junction was derived from the transmural (transwall) pressure value, which is higher in the presence of a higher blood pressure. The details can be found in our published work (11).

### Patient-specific model for transient simulation

**Figure 2:**
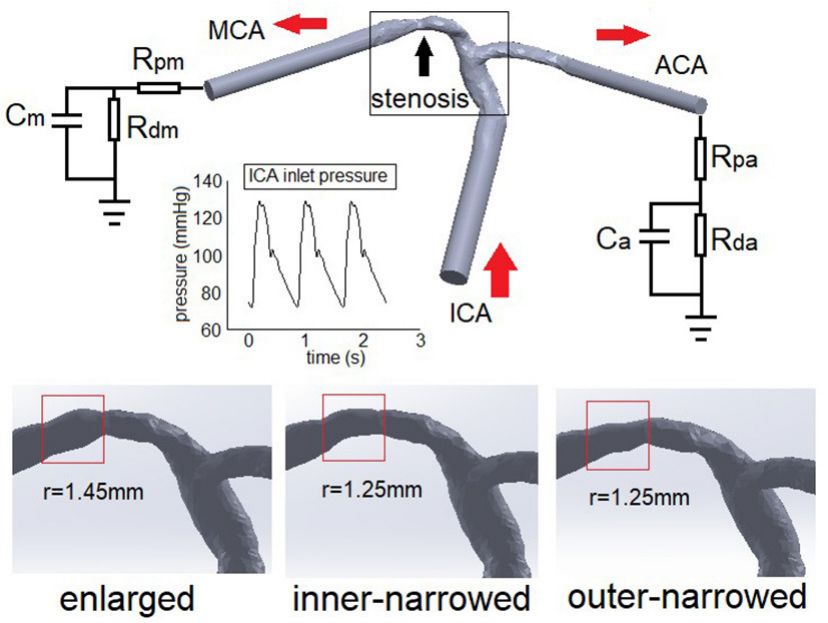
The Windkessel model applied in a patient-specific ICAS model (upper panel) and arterial geometry simulated with different stent shapes (lower panel). The red arrows show the blood flow directions. ICA, internal carotid artery; MCA: middle cerebral artery; ACA, anterior cerebral artery. R, C, p, d, m, a in the Windkessel elements denote resistance, capacitance, proximal, distal, MCA, and ACA, respectively.

Transient simulation was conducted in another patient with a symptomatic ICAS lesion (70% luminal stenosis) at M1 segment of MCA (black arrow in Figure 2), covering the arterial segments of ICA, MCA and ACA as described above. To get fully developed flow in the transient simulation, at each inlet and outlet, the cross-section was lofted into a circle with an identical area, and elongated to form a cylinder with a length of 6 times of the cylindrical diameter (Figure 2). The geometry variations (i.e., enlarged, inner-narrowed, outer-narrowed) were similarly built as in the patient-specific models for static simulation.

Meshing was conducted similarly as above. Pulsatile pressure from physiological measurement was imposed at ICA inlet (23). At each outlet, we applied a Windkessel model consisting of proximal (Rp) and distant (Rd) flow resistances and a capacitance (C) to simulate the elastic arterial wall in the distal segments. The capacitances of MCA and ACA were 1.16 and 0.82 (unit: 10^−10^ m^3^ Pa^−1^). The total resistance of MCA and ACA were 5.97 and 8.48 (unit: 10^9^ Pa s m^−3^), distributed according to Rp/Rd=1/9 based on physiological measurements in intracranial arteries (24). Other conditions were identical with static models. Considering the initial effects, the simulation lasted for three cardiac cycles (0.8 s each). Data of the second cycle were analyzed.

In transient simulation, we investigated the temporal fluctuations of the area-averaged WSS and LDL filtration rate, as well as the ratio of area-averaged WSS values obtained at the outer and inner sides of the stented segment, throughout the whole arterial model.

## Results

### Static models

**Figure 3:**
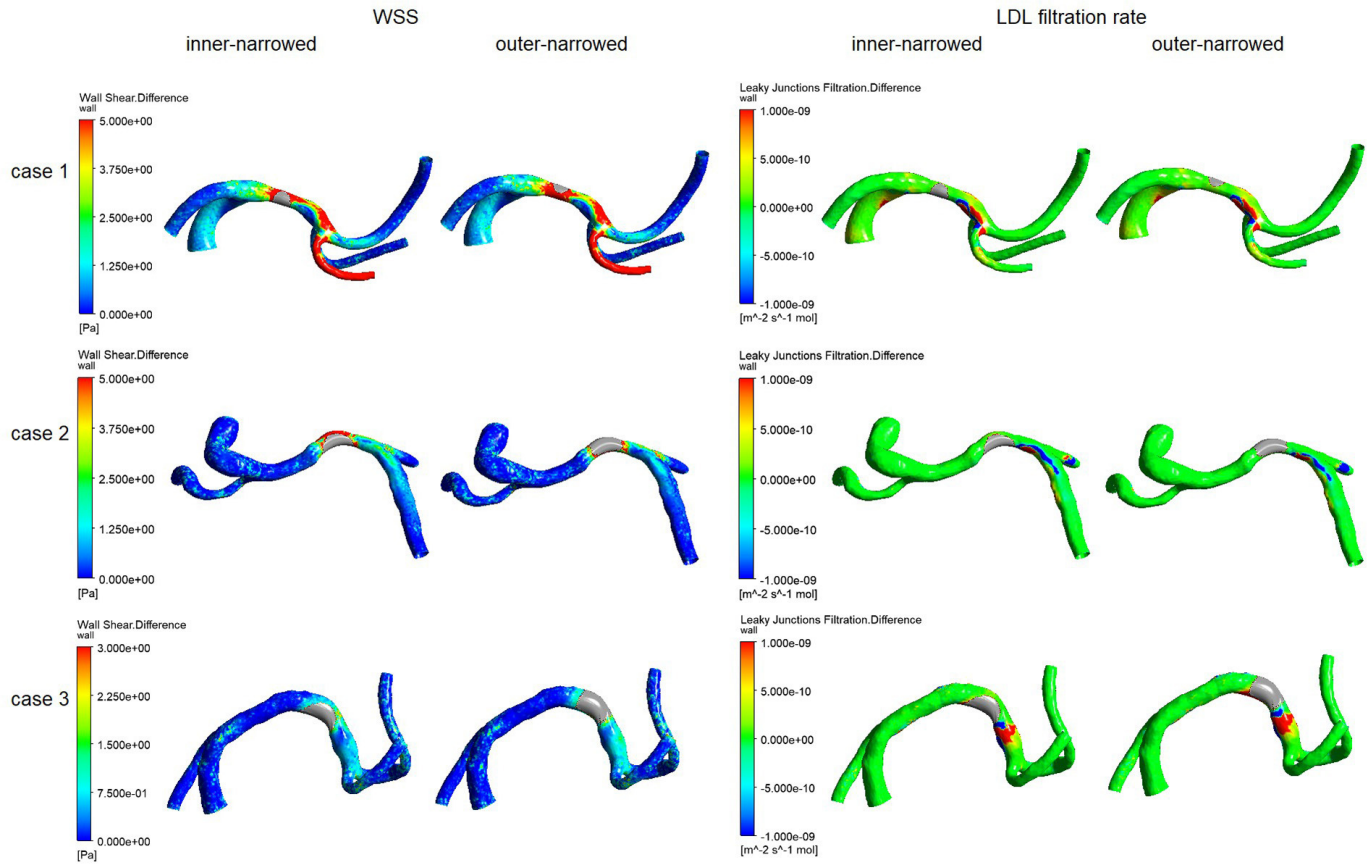
The distribution of differences of WSS and LDL filtration rate, of the inner-narrowed and outer-narrowed models compared with the enlarged model, in patient-specific static models. A red color indicates a larger difference and blue or green color indicates a smaller difference, in the parameter of the inner-narrowed or outer-narrowed model against the enlarged model (as a reference model that is not shown in the figure). For instance, there is larger difference in WSS in the stented segment and an immediate branch (in red color), but smaller difference in WSS in other regions (blue color), in the inner-narrowed model in case 1 in the left panel, as compared with WSS in the enlarged model.

Figure 3 illustrates the spatial distribution of the differences in WSS and LDL filtration rate, in the inner-narrowed and outer-narrowed stent models compared with the enlarged model. The areas proximal to the stent were not much affected. High WSS differences extended around the stented area and in some distal segments, whereas high differences in LDL filtration rate appeared sporadically at certain specks. There were minimal differences in the pressure values among the models that are marginally, visually indifferentiable, which are hence not illustrated in the figure.

**Figure 4:**
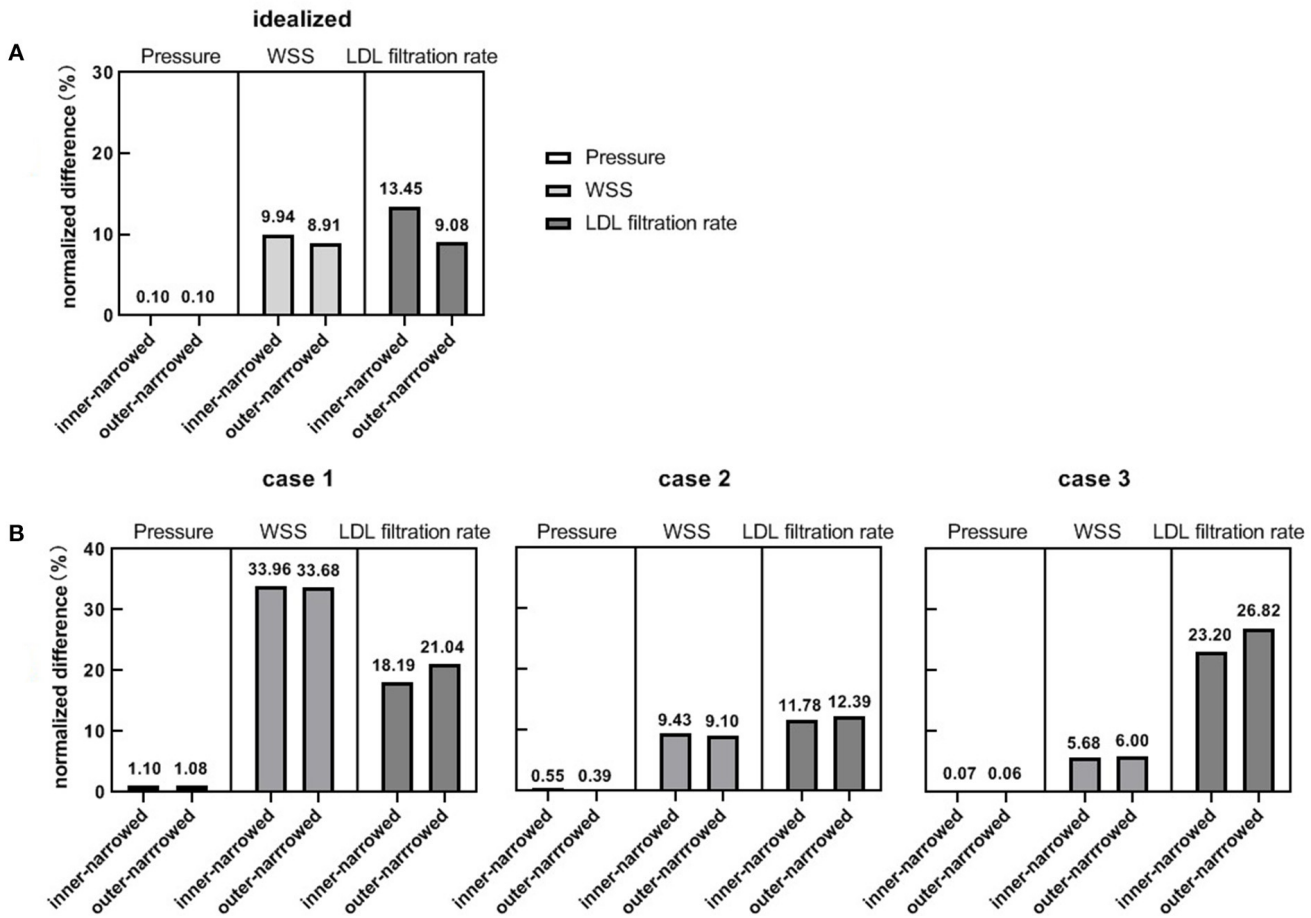
The normalized differences of pressure, WSS and LDL filtration rate, in the inner-narrowed and outer-narrowed models compared with the enlarged model, in the idealized and patient-specific static CFD models. **(A)** Results in idealized model. **(B)** Results in patient-specific models.

Figure 4 shows the NDs of pressure, WSS and LDL filtration rate of the inner-narrowed and outer-narrowed models compared with the enlarged model. The NDs of pressure were within 1% in all scenarios, which was negligible. The NDs of WSS was over 30% in case 1 but below 10% in other two cases. The difference between inner-narrowed and outer-narrowed models in ND of WSS was <2% in all cases. The ND of LDL filtration rate reached 26.8% in the outer-narrowed model of case 3. The difference between inner-narrowed and outer-narrowed models in ND of LDL filtration rate was 4.4% in the idealized model and <4% in the three patient-specific models.

Table 1 shows the ratios of area-averaged WSS values obtained at the outer and inner sides of the stented segment in different cases. In general, the value of this metric was the highest in the enlarged model or the inner-narrowed model, in the idealized model and the 3 cases. The inconsistent findings across these cases were probably due to the different curvature of the stented arterial segment, which can be visualized in Figure 1.

**Table 1:** The ratio of area-averaged WSS values obtained at the outer and inner sides of the stented segment.

**Simulation Type**	**Case or cardiac phase**	**Stent geometry variations**		
		Enlarged	Inner-narrowed	Outer-narrowed
Static	Idealized	1.70	1.65	1.66
	Case 1	1.13	1.27	1.19
	Case 2	0.83	1.03	1.02
	Case 3	1.86	1.56	1.61
Transient	Diastolic	1.88	1.52	1.57
	Systolic	2.06	1.52	1.72

### Transient models

**Figure 5:**
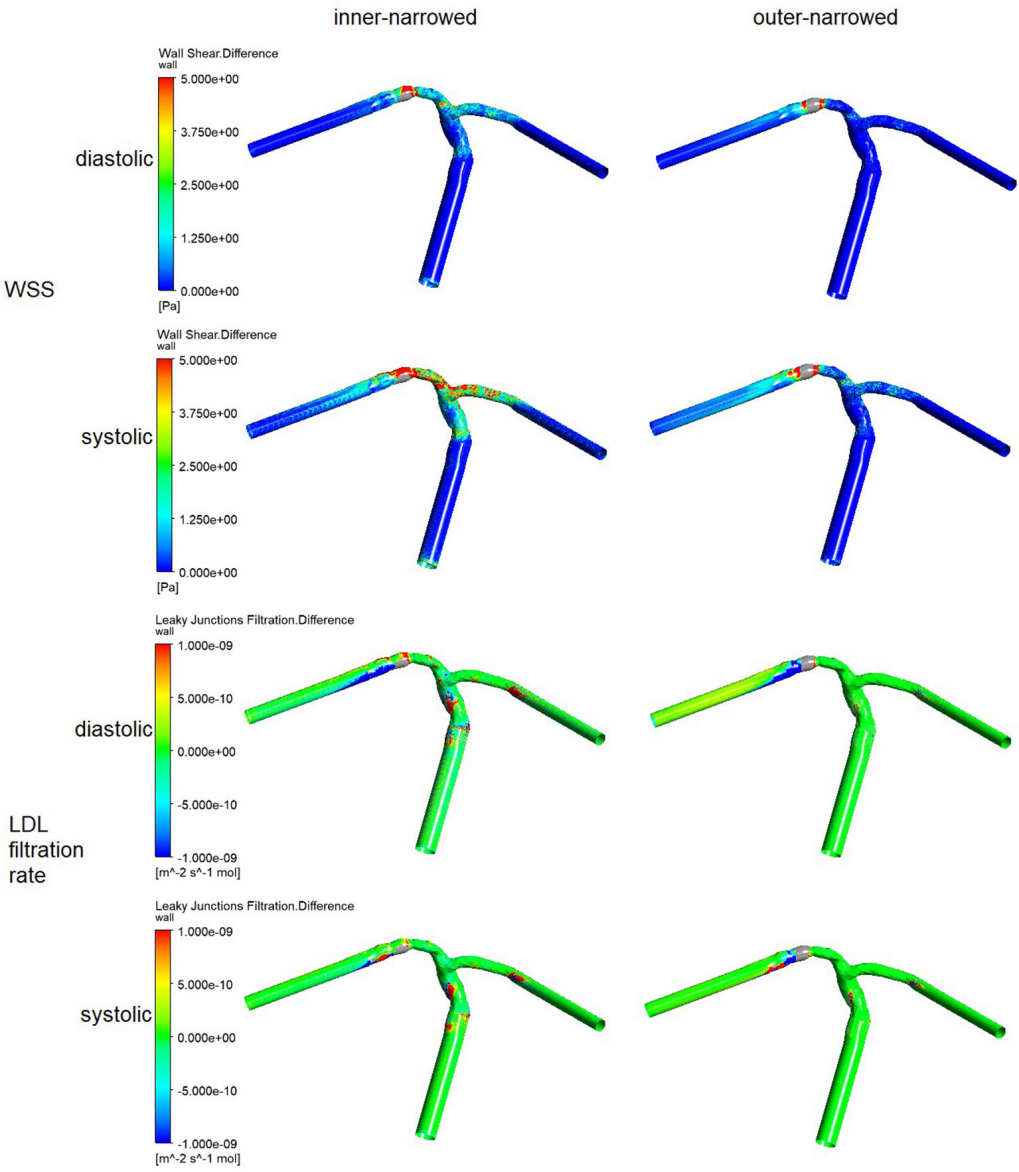
The distribution of the differences in WSS and LDL filtration rate, of the inner-narrowed and outer-narrowed models compared with the enlarged model. The data were measured end-of-diastole and systole of the second cardiac cycle (0.86 s and 1.00 s in simulation) where the inlet blood pressure was minimal and maximal.

Similar with the results from static models (Figure 3), higher differences in WSS with inner- or outer-narrowed stent shapes vs. the enlarged stent shape appeared mainly in the stent area and immediately distal segment. Observable differences also existed in some areas proximal to the stent and even in ACA, particularly with the inner-narrowed stent shape (Figure 5). Also similar with the static models, observable differences in LDL filtration rate with inner- or outer-narrowed vs. the enlarged stent shape appeared sporadically at certain specks. The differences in WSS and LDL filtration rate were more significant in the systolic than diastolic phase. The differences in pressure values among the models are negligible therefore not illustrated in the figure.

**Figure 6:**
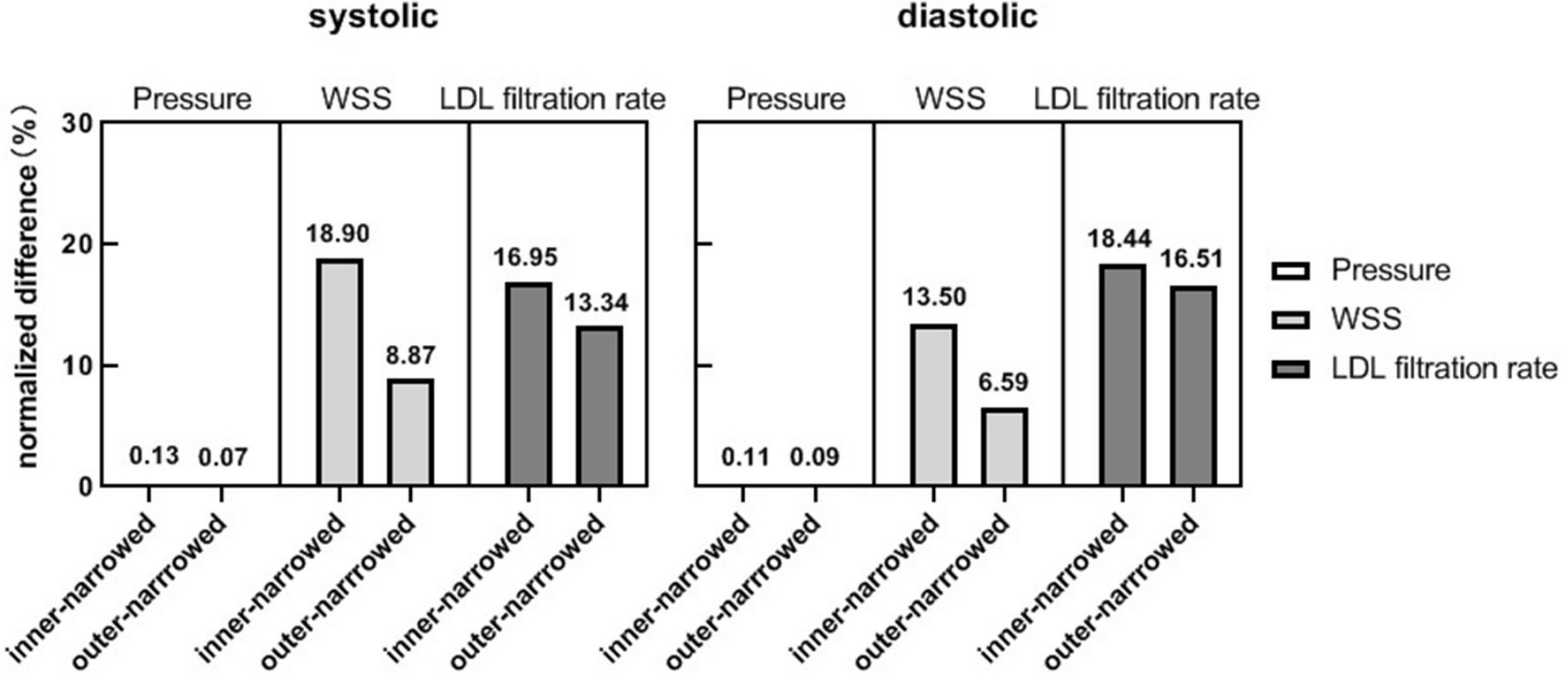
The normalized differences of pressure, WSS and LDL filtration rate, of the inner-narrowed and outer-narrowed models compared with the enlarged model. The data were measured at end-of-diastole and systole of the second cardiac cycle (0.86 s and 1.00 s in simulation) where the inlet blood pressure was minimal and maximal.

As in the static models, the NDs of pressure were negligible in the systolic and diastolic phases of the inner-narrowed and outer-narrowed models (<1% for all). The NDs of LDL filtration rate were comparable with the corresponding value of WSS in the systolic phase, but the former were larger in the diastolic phase (Figure 6).

**Figure 7:**
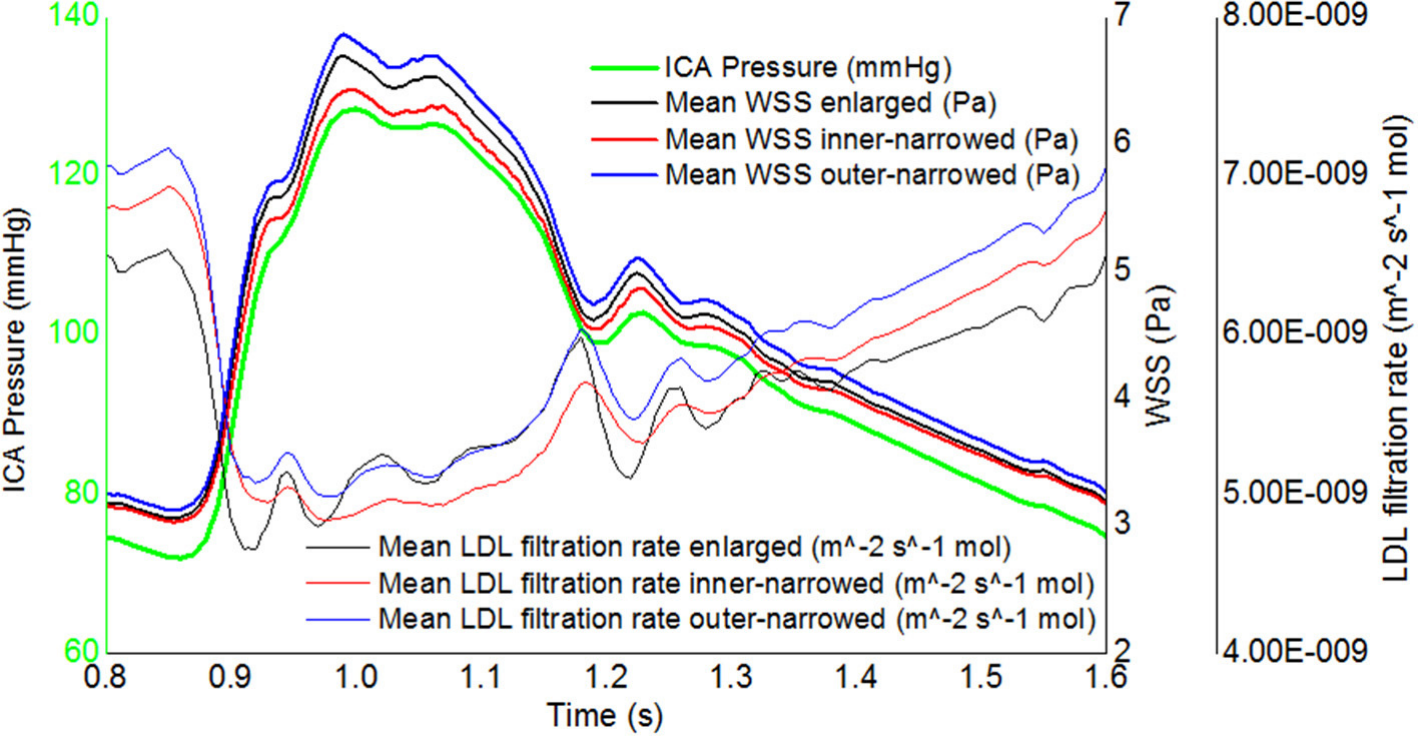
The transient, area-averaged WSS and LDL filtration rate and inlet pressure during the second cardiac cycle.

Regarding the temporal fluctuations, the WSS fluctuations approximately follow the inlet pressure waveform while the fluctuations of LDL filtration rate were oppositely, non-linearly related with the inlet pressure waveform, in three stent shapes (Figure 7). The difference between maximal and minimal LDL filtration rate in a cardiac cycle was less than that of WSS.

Regarding the distribution of WSS in the outer and inner sides of the stented segment, the ratio of area-averaged WSS obtained at these two areas was larger in the enlarged model than the other two models, in the diastolic and systolic phases (Table 1).

## Discussion

In this preliminary study, based on idealized and patient-specific ICAS models, we investigated the effects of different stent geometry on focal hemodynamic parameters adjacent to the stenotic lesion, with manually manipulated and simulated stent shapes. We found that the effect of stent geometry on focal pressure is negligible, but there were potential differences in the WSS and LDL filtration rate between stent shapes, with normalized difference exceeding 20% in certain cases. Observable stent-induced differences in WSS and LDL filtration rate mainly appeared around the stented area and in immediately distal segments, which could also exist in more distal branches. To our knowledge, this is among the first attempts to non-invasively simulate and evaluate the effect of stent geometry on focal hemodynamics in patients with ICAS.

Stenting currently is not a first-line treatment for patients with symptomatic ICAS, with higher stroke and mortality rates compared with medical treatment alone in the Stenting and Aggressive Medical Management for Preventing Recurrent Stroke in Intracranial Stenosis (SAMMPRIS) trial published in 2011 (25), and similar stroke and mortality rates with medical treatment in the most recent China Angioplasty and Stenting for Symptomatic Intracranial Severe Stenosis (CASSISS) trial (26). In addition to the concern over peri-procedural complications, which has been significantly reduced in recent studies with refined patient eligibility criteria and more experienced interventionalists, ISR is also a concern or a marker of treatment failure. In carotid artery studies, stent geometry plays a pivotal role in the development of ISR (27). However, geometry parameters alone are not sufficient to directly associate with restenosis, while investigations from a hemodynamic perspective may help explain the relationships of stent geometric features with the risk of ISR (28). Previous CFD studies in stenosis of other arteries (mostly coronary and carotid arteries) indicated that WSS, its gradient, and its temporal fluctuations are indicative of ISR (5, 28–30). In accordance with existing studies, our results suggest that the stent geometry can considerably influence WSS, which is observable even in some downstream branches while the areas adjacent to the stent are the most significantly affected. Moreover, the inconsistent findings on the distribution of WSS at the outer and inner sides of the stented segment in these cases have also indicated the role of the arterial geometry (e.g., curvature) in affecting focal hemodynamics, in addition to the stent shapes, as indicated in a previous study mentioned above (6). Therefore, both the stent shape and the arterial geometry (in the stented and proximal segments) warrant consideration in future studies on hemodynamic changes after stenting in ICAS.

In addition to WSS, we also evaluated the parameter of LDL filtration rate, which was not investigated in most existing studies of intracranial arteries. Physiologically, the LDL filtration rate could be affected by multiple hemodynamic factors including transmural pressure and WSS. Therefore, compared with WSS, investigating the associations between different stent shapes and LDL filtration rates could more comprehensively reflect the hemodynamic effects of the stents on the endothelium, which is more directly related with further development of atherosclerosis and ISR. However, it had not been studied in the stenting of ICAS cases. Our research fills this gap, where the results showed significant changes in the LDL filtration rate with different stent shapes in ICAS.

The LDL filtration rate obtained in intracranial arteries in this study was in the same order of magnitude with previous studies in coronary arteries (31). However, *in vivo* it may be slightly different in different arterial beds. Some important factors that may affect LDL filtration rate were not modeled in our investigations. For instance, the histology of the intracranial arterial wall has differences with extracranial arteries, with a denser internal elastic lamina and without an external elastic lamina (32), which could affect the LDL permeation rate. Additionally, the filtration of LDL into arterial wall is only among the initial steps of atherogenesis which is a complex process with many other factors involved. Thus, the LDL filtration rate could indicatively but not quantitatively reflect the risk of ISR in intracranial arteries. In-depth pathological investigations are needed to verity and optimize the 3-pore model to achieve more reliable evaluation of the LDL filtration rate, and longitudinal studies are needed to reveal the associations between LDL filtration rate and the risk of ISR in patients with ICAS.

Of note, the LDL filtration rate had milder temporal fluctuations than WSS in a cardiac cycle (Figure 7). Computational algorithms of the 3-pore model could partly explain this phenomenon. The LDL filtration rate per one leaky junction is positively related to the transmural pressure gradient, but the density of leaky junctions is negatively related to WSS. During the systole, the high blood pressure leads to a high transmural pressure which accelerates the transport of LDL molecule across the endothelium (increased LDL filtration rate per one leaky junction), while the high flow rate leads to a high WSS which decreases the density of leaky junctions. During the diastole, the low WSS increases the density of leaky junctions whilst the low blood pressure decreases the LDL filtration rate per one leaky junction. Therefore, the effects of pressure and WSS fluctuations are to some extent “neutralized” in LDL filtration rate. This observation indicated that in addition to the investigations of WSS in hemodynamic studies of ICAS as in most previous studies, the LDL filtration rate needs to be evaluated independently for its effects. Meanwhile, in the simplified 3-pore model, the LDL filtration rate was calculated from local hemodynamic parameters while other parameters (e.g., the permeability of the endothelium or the plaque surface) were considered as constants, as in previous relevant studies (6, 11, 33). Currently we lack patient-specific values of these parameters, which need to be explored in future studies.

There were some limitations in this study. Firstly, this is a small-scale pilot study which includes only 3 patient-specific models with ICAS at MCA in static simulation. This may lead to “accidental error” in the results. Large-scale validations are needed to further investigate the credible range of the stent-induced changes in focal hemodynamics in patients with ICAS at various locations, and to take into account the effects of various geometric features of cerebral arteries and the ICAS lesions on the findings. Secondly, the simulated geometry of the stents was spatially simplified; the three variants of stent geometry were based solely on the caliber instead of explicit stent structures, while the curvature and dents around the verges of a stent could also lead to changes in focal hemodynamics. In addition, a solid wall assumption was used without considering the plaque composition and elasticity of the arterial wall, while the arterial wall compliance and plaque composition can influence the local WSS estimation especially in transient simulation (34). Therefore, fluid-solid interaction (FSI) simulation with a refined 3-pore model could be introduced in future models to reveal the possible differences in LDL filtration rates in intracranial vs. extracranial arteries as discussed above. Regarding the evaluation of the hemodynamic parameters, we used the globally area-averaged values, but focal distributions and variations of these parameters warrant further investigations to associate with or predict the possible locations of plaque growth or restenosis after stenting. Last but not least, the boundary conditions including ICA pressure and the parameters of the Windkessel and 3-pore models were derived from literature instead of patient-specific measurements. Although introducing patient-specific boundary conditions (e.g., based on patient-specific blood pressure and flow velocities) in the models would confound the analyses in this small-scale, pilot study, such patient-specific boundary conditions could be tested in future larger-scale, longitudinal studies, to validate our findings and further reveal the relationships of arterial and stent geometry, focal hemodynamics and plaque growth after stenting.

## Conclusions

In this preliminary study, based on idealized and patient-specific ICAS models, we found that the stent shape might influence the WSS and LDL filtration rate in ICAS, which mainly appeared in area adjacent to and immediately distal to the stent. Future studies are warranted to verify the relevance of the changes in these hemodynamic parameters in governing plaque growth and ISR after stenting in ICAS.

## Data availability statement

The original contributions presented in the study are included in the article/supplementary material, further inquiries can be directed to the corresponding author.

## Ethics statement

The studies involving human participants were reviewed and approved by the Joint Chinese University of Hong Kong—New Territories East Cluster Clinical Research Ethics Committee (The Joint CUHK-NTEC CREC). The patients/participants provided their written informed consent to participate in this study.

## Author contributions

HL and XL contributed to conception, design of the study, and organized the database. HL performed the statistical analysis and wrote the first draft of the manuscript. HL, YL, and XL wrote sections of the manuscript. All authors contributed to manuscript revision, read, and approved the submitted version.
